# Lyoniresinol Improves Insulin Sensitivity and Induces White-to-Beige Adipose Tissue Conversion in Obese Rats: Associated with JNK/PI3K/mTOR Pathway Modulation

**DOI:** 10.5812/ijpr-171105

**Published:** 2026-08-19

**Authors:** Wen Lei, Alok Tripathi, Shuwen Hu

**Affiliations:** 1 Department of Endocrinology, Xi'an Hospital of Traditional Chinese Medicine, Xi'an 710021, Shaanxi, China; 2 Department of Pharmacology, Era College of Pharmacy, Era University, Lucknow, Uttar Pradesh, India; 3 Department of Endocrinology and Genetic Metabolism, Xi'an Children's Hospital, The Affiliated Children's Hospital of Xi'an Jiaotong University, Xi'an, Shaanxi, China

**Keywords:** Lyoniresinol, Obesity, Insulin Sensitivity, JNK/PI3K/mTOR Pathway, White Adipose Tissue Browning

## Abstract

**Background:**

Obesity contributes to multiple chronic diseases and remains a critical global health challenge.

**Objectives:**

This study evaluated the therapeutic effects of lyoniresinol on high-fat diet-induced obesity and its underlying molecular mechanisms.

**Methods:**

Network pharmacology was used to identify potential targets of lyoniresinol in obesity treatment, followed by validation using molecular docking. High-fat diet-induced obese rats received lyoniresinol (30 mg/kg/day) for six weeks. The primary efficacy endpoints were insulin sensitivity, assessed by the oral glucose tolerance test (OGTT), insulin tolerance test (ITT), fasting glucose, and fasting insulin, and adiposity measures, including body weight and regional fat mass. The key secondary endpoint was uncoupling protein 1 (UCP1) expression in adipose tissue, evaluated by Western blotting and immunohistochemistry. Mechanistic validation comprised analysis of the phosphorylation status of c-Jun N-terminal kinase (JNK), phosphatidylinositol 3-kinase (PI3K), and mammalian target of rapamycin (mTOR) by Western blotting. Exploratory assessments included serum lipid profiles, inflammatory cytokines, oxidative stress biomarkers, and histopathological examination.

**Results:**

Network pharmacology identified 50 shared targets between lyoniresinol and obesity. Molecular docking indicated favorable binding to JNK, PI3K, and mTOR. For the primary endpoints, lyoniresinol treatment was associated with reduced body weight gain, restored normoglycaemia, enhanced insulin sensitivity, and decreased regional adipose tissue mass. For the key secondary endpoint, UCP1 expression was upregulated, suggesting the browning of white adipose tissue. For the exploratory endpoints, lyoniresinol was associated with improved dyslipidaemia, reduced oxidative stress, suppressed pro-inflammatory cytokines and C-reactive protein (CRP), and attenuated adipocyte hypertrophy. For mechanistic validation, lyoniresinol treatment was associated with suppressed JNK phosphorylation and activation of PI3K/mTOR signaling in adipose tissue, suggesting potential involvement of the JNK/PI3K/mTOR pathway in these effects.

**Conclusions:**

Based on the primary efficacy endpoints, lyoniresinol was associated with improved insulin sensitivity and reduced adiposity in obese rats. The key secondary endpoint (UCP1 upregulation) and mechanistic validation (altered JNK/PI3K/mTOR phosphorylation) further support its association with the browning of white adipose tissue. These findings suggest that lyoniresinol may represent a multi-target candidate for further investigation in obesity-related metabolic disorders; however, the precise molecular mechanisms require additional validation.

## 1. Background

Obesity is a metabolic condition in which expansion of the body’s fat storage in adipose tissue disrupts the balance between energy intake and energy expenditure (1). It is a major risk factor for many chronic conditions, including type 2 diabetes. Obesity alters insulin sensitivity because inflammatory adipokines secreted by adipose tissue modulate metabolic pathways involved in lipid deposition and promote the transdifferentiation of brown adipose tissue (BAT) into white adipose tissue (WAT) (2). Obesity-induced inflammation and oxidative stress in adipose tissue impair insulin signaling and promote insulin resistance (3). Similar findings have been reported with dill seed extract combined with endurance training in obese rats (4). Adipose tissue remodeling contributes to the development of insulin resistance, ultimately leading to type 2 diabetes. Conventional pharmacological approaches to obesity have limitations, highlighting the need for additional therapeutic interventions.

The PI3K/AKT signaling pathway is involved in the pathogenesis of obesity. AKT activation is also associated with the regulation of cell proliferation and growth and has implications for obesity development (5). Growth factors and hormones can activate PI3K and AKT (6). PIP2 is converted to PIP3, which activates downstream proteins (FoxO, PKCs, and GSK3), thereby regulating adipogenesis, glucose uptake, and glycogen synthesis. The PI3K/AKT pathway plays an important role in insulin signaling and metabolic regulation. Impaired PI3K/AKT activation is associated with obesity-induced insulin resistance (7). PI3K/AKT also interacts with mTOR. The PI3K/AKT/mTOR axis is closely related to obesity development (8). This pathway is also associated with thermogenesis, and activation of this signaling axis has been reported to promote weight loss and WAT browning (9). The literature has also shown that PI3K/AKT signaling downregulation is associated with high-fat diet (HFD)-induced obesity and that glutamine supplementation can maintain the metabolic function of HFD-fed rats through modulation of PI3K/AKT (5). This signaling pathway is a promising target for combating obesity.

Lignan compounds exhibit antioxidant and metabolic regulatory activities and may improve glucose and lipid metabolism through modulation of oxidative stress and insulin signaling pathways (10). Lyoniresinol is a lignan compound extracted from several medicinal plants, such as *Acanthus ilicifolius*, *Toona sinensis*, and *Berberis vulgaris* (11). Lyoniresinol has demonstrated cytoprotective and antioxidant effects (12). Moreover, lyoniresinol has been reported to have anti-melanogenic activity and a skin-lightening effect by activating extracellular signal-regulated kinase (ERK), which inhibits tyrosinase expression (13). Recently, lyoniresinol was shown to exert a protective effect against cerebral ischemic stroke injury by regulating the AKT/GSK-3β/Nrf2 signaling pathway via suppression of oxidative stress (14). Because AKT signaling is closely involved in insulin action and metabolism, regulation of AKT-related pathways by lyoniresinol suggests its potential to improve insulin sensitivity. However, the effects of lyoniresinol on obesity-associated insulin resistance remain unclear.

## 2. Objectives

Therefore, we hypothesized that lyoniresinol may ameliorate obesity-associated insulin resistance by modulating AKT-related signaling pathways.

## 3. Methods

### 3.1. Network Pharmacology Analysis

The chemical structure of lyoniresinol (PubChem CID: 11711453, CAS number: 14464 - 90 - 5, molecular weight: 420.5 g/mol) was obtained from the PubChem database. Potential targets of lyoniresinol were predicted using the SwissTargetPrediction database (http://www.swisstargetprediction.ch), specifying *Homo sapiens* and applying a probability threshold of ≥ 0.1. Genes associated with obesity were retrieved from the DisGeNET database. Common targets were defined as genes present in both the lyoniresinol target list and the obesity-associated gene list. Overlapping targets between lyoniresinol and obesity were identified using Venn diagram analysis. Subsequently, a protein-protein interaction (PPI) network was constructed using STRING database (https://string-db.org) version 12.0, limited to *Homo sapiens*, with a medium-confidence interaction score threshold of ≥ 0.4. This threshold was selected to capture biologically meaningful interactions while minimizing false-positive associations. Network topological parameters (nodes, edges, average node degree, and clustering coefficient) were extracted directly from the STRING output. Gene Ontology (GO) and Kyoto Encyclopedia of Genes and Genomes (KEGG) pathway enrichment analyses were performed for the overlapping targets using the SRplot online platform (https://www.bioinformatics.com.cn/srplot), with significance set at P < 0.05 after Benjamini-Hochberg correction. All computational predictions were considered hypothesis-generating and required subsequent experimental validation.

### 3.2. Molecular Docking Analysis of Lyoniresinol With mTOR, PI3K, and JNK

The 3D molecular structure of lyoniresinol was downloaded from the PubChem database (https://pubchem.ncbi.nlm.nih.gov/). The crystal structures of JNK (PDB ID: 1UKH), PI3K (PDB ID: 5DXT), and mTOR (PDB ID: 9F42) were downloaded from the Protein Data Bank (PDB) (http://www.rcsb.org/pdb). These proteins were selected as docking targets because they were identified as key nodes in the PPI network and have been reported to be involved in adipocyte browning and insulin signaling. Protein structures were prepared using Discovery Studio Visualizer v20.1.0.19295 (Dassault Systems, San Diego, CA, USA). Water molecules and unnecessary heteroatoms were removed before docking. Molecular docking was performed using AutoDock Vina v1.1.2. Docking grids were generated based on the reported active sites of the target proteins. Binding affinity scores and interaction patterns were analyzed. Binding affinity values below -5.0 kcal/mol were considered indicative of favorable ligand-protein interactions based on previously reported criteria. The predicted binding modes were further evaluated by in vivo experiments.

### 3.3. In Vivo Study

#### 3.3.1. Animals

Male Wistar rats (165 - 190 g) in good health were maintained under specific pathogen-free conditions (humidity 55 ± 5%, temperature 25 ± 2 °C) with a 12-hour light/dark cycle at Sant Gadge Baba Amravati University. All animal studies were approved by the Institutional Ethical Committee of Sant Gadge Baba Amravati University (Approval No. 650/02/C/CPCSEA/04/2022). All procedures complied with the CPCSEA guidelines for the care and use of laboratory animals. During the experiment, animals were monitored daily for signs of pain or distress. No analgesics were required because the procedures did not cause more than momentary pain. All animals were euthanized by cervical dislocation under isoflurane anesthesia at the end of the study.

#### 3.3.2. Obese Rat Model Construction and Treatment

A total of 24 Wistar rats were randomly divided into four groups (n = 6 per group) using a computer-generated random number sequence: the control group (received a normal diet), the obese group (received HFD with vehicle), the lyoniresinol-treated group (received HFD along with 30 mg/kg lyoniresinol orally), and the standard (STD) group (received HFD along with 40 mg/kg atorvastatin orally). Investigators were blinded to group allocation during both interventions and outcome assessment using coded oral gavage syringes prepared by an independent researcher. Obesity was induced by feeding rats an HFD for six weeks based on a previously described method (12). The normal control diet contained 64% carbohydrate, 24% protein, and 12% fat, providing 318.8 kcal/100 g (3.188 kcal/g). The HFD contained 31% fat, 12% protein, and 46% carbohydrate, with an energy density of 516.5 kcal/100 g (5.165 kcal/g). Lyoniresinol (30 mg/kg) and atorvastatin (40 mg/kg) were suspended in vehicle (0.5% carboxymethylcellulose, in sterile water) and administered daily by oral gavage at 9:00 AM for six consecutive weeks. Atorvastatin was used as a positive control because it is a widely prescribed lipid-lowering agent that improves obesity-related metabolic disturbances. The dosing volume was 5 mL/kg body weight, and the volume was adjusted weekly based on individual body weight measurements to maintain the target dose. The control and obese groups received an equal volume of vehicle alone on the same schedule. All animals that completed the 6-week experimental protocol were included in the final analysis, and no animals were excluded or replaced.

#### 3.3.3. Insulin Tolerance and Glucose Tests (Primary Efficacy Endpoint)

The insulin tolerance test and glucose tolerance test were performed at week 6 of the intervention period. For the oral glucose tolerance test (OGTT), rats were fasted for 6 hours before testing. Glucose was administered orally at 2 g/kg body weight, and blood samples were collected from the retro-orbital sinus using heparinized capillary tubes at 0, 30, 60, 90, and 120 minutes after administration. Blood glucose levels were determined immediately using an autoanalyzer (Mindray BS-240, China). For the insulin tolerance test (ITT), human regular insulin (Novolin R, Novo Nordisk) was injected intraperitoneally at 0.75 U/kg body weight after a 4-hour fast. Blood glucose was measured from tail vein samples at 0, 30, 60, 90, and 120 minutes after insulin injection using the same autoanalyzer.

#### 3.3.4. Lipid Profile (Exploratory Endpoint)

Serum total cholesterol, triglycerides, high-density lipoprotein (HDL), and low-density lipoprotein (LDL) were measured using an autoanalyzer (Mindray BS-240) with commercial kits (BioSino, China), according to the manufacturer’s protocol. Each sample was tested in duplicate.

#### 3.3.5. Inflammatory Cytokines and C-Reactive Protein (Exploratory Endpoint)

Serum concentrations of inflammatory cytokines (IL-1β, IL-6, IL-17, and TNF-α) and C-reactive protein (CRP) were measured using ELISA kits (Elabscience, China), according to the manufacturer’s instructions. Absorbance was read at 450 nm using a microplate reader (Bio-Tek, USA). Each sample was tested in duplicate, and cytokine concentrations were calculated from standard curves.

#### 3.3.6. Assessment of Oxidative Stress (Exploratory Endpoint)

Oxidative stress parameters, including malondialdehyde (MDA), superoxide dismutase (SOD), and glutathione (GSH), were measured in serum samples. MDA levels were determined using the thiobarbituric acid reactive substances (TBARS) method, with modifications. Briefly, 100 μL of serum was mixed with 0.2 mL of 8.1% sodium dodecyl sulfate, 1.5 mL of 20% acetic acid (pH 3.5), and 1.5 mL of 0.8% thiobarbituric acid. The mixture was heated at 95°C for 60 minutes, cooled, and extracted with n-butanol-pyridine (15:1, v/v). The absorbance of the organic phase was measured at 532 nm using a spectrophotometer (UV-1800, Shimadzu, Japan). SOD activity was determined by the epinephrine method according to Misra and Fridovich. Serum samples were diluted 1:10 and reacted with 0.05 M sodium carbonate buffer (pH 10.0) and 0.03 M epinephrine, and the rate of epinephrine autoxidation was monitored at 480 nm for 3 minutes at 25°C. One unit of SOD activity was defined as the amount of enzyme required to inhibit 50% of epinephrine autoxidation. GSH activity was measured using t-butyl hydroperoxide as a substrate according to Flohe and Gunzler. The reaction mixture (1.0 mL final volume) contained 0.1 M phosphate buffer (pH 7.0), 1 mM EDTA, 1 mM sodium azide, 1 mM reduced GSH, 0.2 mM NADPH, and 0.5 U/mL glutathione reductase. The reaction was carried out at 37°C. Briefly, 20 µL of serum sample was added to the reaction mixture, and the reaction was initiated by adding 0.2 mM t-butyl hydroperoxide. The decrease in absorbance at 340 nm was monitored for 3 minutes using a UV-Vis spectrophotometer (Shimadzu UV-1800, Japan). GSH activity was calculated using the molar extinction coefficient of NADPH (6.22 × 10^3^ M/cm). One unit of enzyme activity was defined as 1 µmol of NADPH oxidized per minute per milliliter of serum. All measurements were performed in duplicate.

#### 3.3.7. Histopathological Analysis (Exploratory Endpoint)

Adipose depots from all rats were harvested at the end of the experiment after euthanasia by cervical dislocation. Each rat was placed in the supine position, incisions were made to expose the adipose depots, and fat from the intraperitoneal, epididymal, mesenteric, and inguinal regions was dissected and weighed separately. After weighing, samples of epididymal adipose tissue were fixed in 10% neutral buffered formalin for 48 hours, then dehydrated in graded ethanol, cleared in xylene, and embedded in paraffin. Sections (5 μm) were cut, mounted on slides, and stained with hematoxylin and eosin according to the manufacturer’s protocols.

#### 3.3.8. Immunohistochemical Analysis (Key Secondary Endpoint)

Adipose tissue was fixed in 4% paraformaldehyde, embedded in paraffin, and cut into 4 μm sections. After deparaffinization and rehydration, antigen retrieval was performed by microwave heating in EDTA buffer (pH 9.0). Endogenous peroxidase activity was blocked with 3% H_2_O_2_. Sections were blocked with 5% normal goat serum and then incubated with an anti-UCP1 primary antibody (Proteintech, 23673 - 1-AP) at 4 °C overnight, followed by an HRP-conjugated secondary antibody (Biosharp, BL003A) at room temperature for 50 min. DAB was used for color development, and sections were counterstained with hematoxylin. Average optical density (AOD) was quantified using Image-Pro Plus software.

#### 3.3.9. Western Blot Analysis (Key Secondary and Mechanistic Validation Endpoints)

Adipose tissue (50 mg) was homogenized in liquid nitrogen and lysed in 500 μL RIPA buffer supplemented with 1 mM PMSF. The lysate was incubated on ice for 30 minutes and centrifuged at 12,000× g for 15 minutes at 4°C. Protein concentration in the supernatant was measured using the BCA assay. Thirty micrograms of protein per sample were resolved on 10% SDS-PAGE and electrotransferred to PVDF membranes. Membranes were blocked with 5% skim milk for 1 hour at room temperature and then probed overnight at 4°C with primary antibodies against PI3K, JNK, mTOR, UCP1, or GAPDH. After three washes with TBST, membranes were incubated with HRP-conjugated secondary antibodies for 1 hour at room temperature. Protein bands were detected using an ECL chemiluminescence reagent and captured on X-ray film. Band intensities were quantified using ImageJ software. Total protein levels of JNK, PI3K, mTOR, and UCP1 were normalized to GAPDH, whereas phosphorylation levels were evaluated by calculating the ratios of phosphorylated proteins to their corresponding total proteins (p-JNK/JNK, p-PI3K/PI3K, and p-mTOR/mTOR).

### 3.4. Statistical Analysis

All analyses were performed using GraphPad Prism version 9.0. Data are presented as mean ± SD. Multiple-group comparisons were conducted using one-way ANOVA followed by Tukey's post hoc test for pairwise comparisons. The F statistic and degrees of freedom (df) were calculated for each ANOVA. Exact p-values were reported for individual comparisons, and P < 0.05 was considered statistically significant.

## 4. Results

### 4.1. Identification of Therapeutic Targets of Lyoniresinol Against Obesity

**Figure 1.**
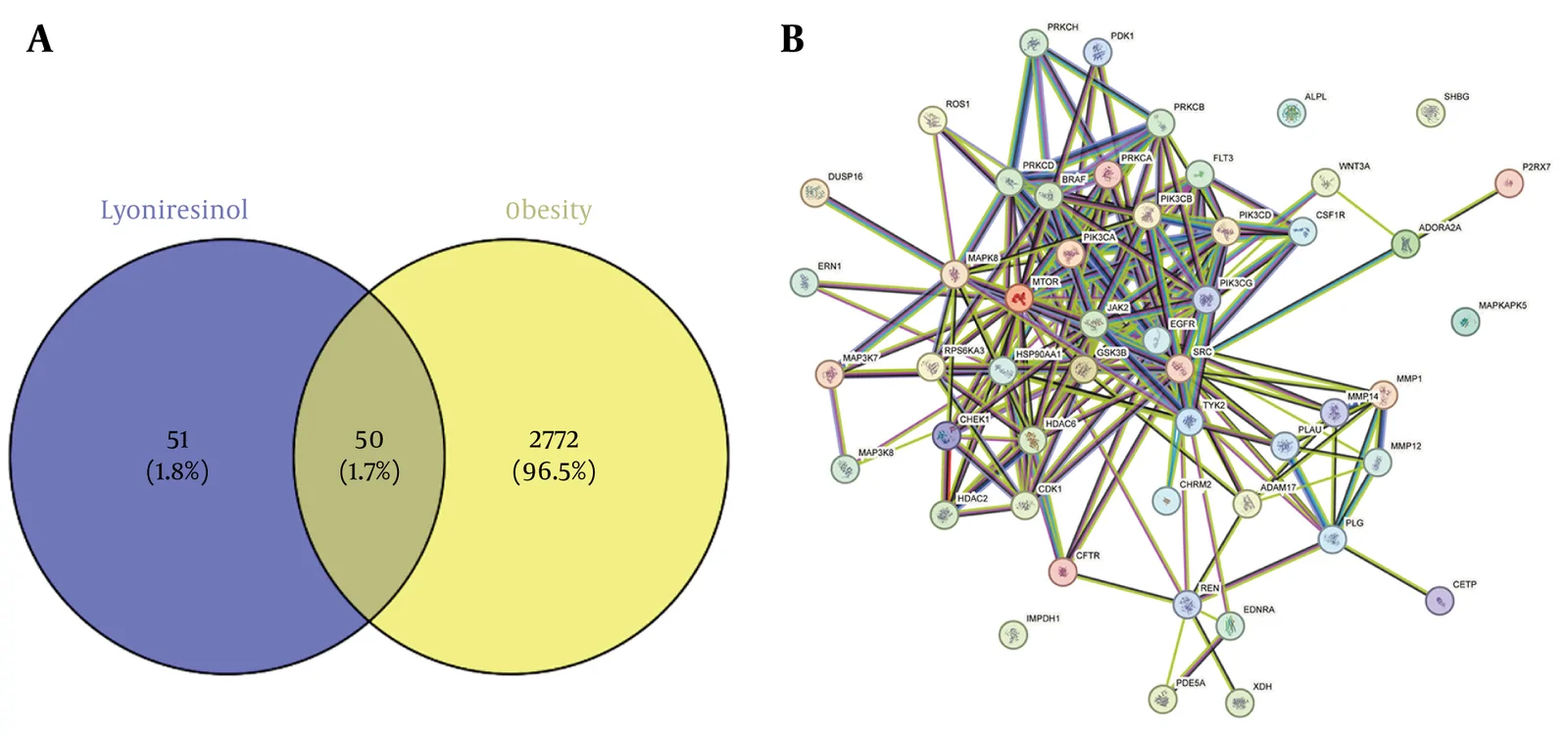
Identification and network analysis of common targets between lyoniresinol and obesity. A: Venn diagram showing the common targets shared by lyoniresinol and obesity. B: Protein-protein interaction (PPI) network of the 50 overlapping genes, constructed using the STRING web server with a confidence threshold of 0.4.

We used a network pharmacology approach to identify therapeutic targets of lyoniresinol for obesity treatment. The structure of lyoniresinol was retrieved from PubChem. SwissTargetPrediction identified 101 targets of lyoniresinol with a probability score ≥ 0.1 (Table 1). DisGeNET yielded 2822 obesity-associated genes. Intersection analysis identified 50 common targets of lyoniresinol for obesity treatment (Figure 1A). These targets were used to construct a PPI network using STRING with a confidence score ≥ 0.4, generating a network of 50 nodes and 221 edges. The average node degree was 8.5, and the clustering coefficient was 0.627 (Figure 1B).

**Table 1.** Predicted Potential Protein Targets of Lyoniresinol Identified Using the SwissTargetPrediction Database

MTOR	CCNE2 CDK2 CCNE1	2MAPK8	MMP13	PRKCG	CNR2
**PIK3CA**	KIT	MAPK10	MAP2K7	JAK2	CDC25C
**GSK3B**	KDR	PIK3CD	ADAM17	PRKCD	CDC25A
**CDK2**	SORD	PIK3CB	RELA	PRKCB	TLR9
**PDE5A**	CFTR	RPS6KA3	WNT3A	PRKCE	EGFR
**ADORA2A**	NTRK1	ROCK2	TNKS2	PRKCH	KCNJ1
**FLT3**	ADCY1	RPS6KA4	SHBG	PRKCQ	CSF1R
**MAP2K2**	PRKCA	MAPK11	P2RX3	MAP3K8	CHRM2
**MAPKAPK5**	P2RX7	CDC42BPA	CDK1	EDNRA	CHRM1
**TYK2**	SRC	CSNK1E	IMPDH1	ERN1	CHRM3
**ACVRL1**	CCNE2 CDK2 CCNE1	NLK	ADK	SF3B3	PNP
**MAP3K14**	KIT	ROS1	XDH	HSP90AA1	PLG
**PIK3CA PIK3R1**	KDR	TOP1	HDAC6	MMP7	PLAU
**CHEK1**	MAP3K7	IRAK4	HDAC2	MMP12	PDK1
**CDC25B**	NUDT1	PIK3CG	HDAC1	MMP8	LRRK2
**WEE1**	HSP90AB1	PSMB2	BRAF	ALPL	HPGD
**CETP**	MMP1	MMP14	JAK1	AKR1B10	REN
**PSEN2 PSENEN NCSTN APH1A PSEN1 APH1B**					

### 4.2. Functional Enrichment Analyses

**Figure 2.**
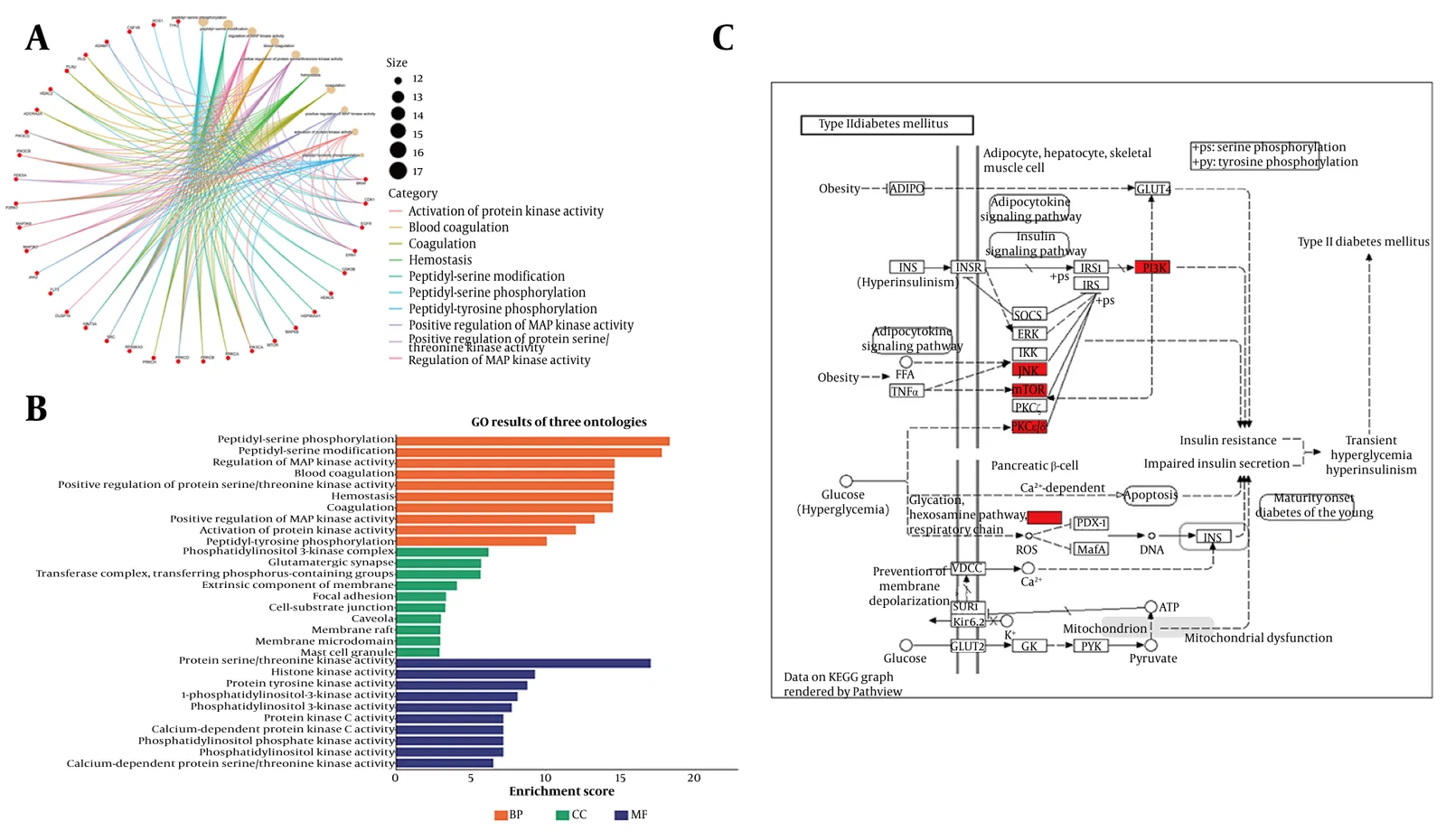
Functional enrichment analysis of lyoniresinol against obesity. A & B: Gene Ontology (GO) analysis of the target proteins of lyoniresinol in treating obesity. C: KEGG pathway analysis showing the involvement of lyoniresinol target proteins in type 2 diabetes-related pathways associated with obesity.

Based on the 50 common targets, GO and KEGG pathway enrichment analyses were performed to elucidate the potential mechanisms of lyoniresinol against obesity. GO enrichment analysis showed that these targets were significantly enriched in the regulation of protein kinase activity, peptidyl-serine phosphorylation, and the positive regulation of MAP kinase activity (Figure 2A-B). Cellular component terms indicated that these targets were predominantly localized to membrane microdomains, focal adhesions, and cell-substrate junctions. Molecular function enrichment highlighted protein serine/threonine kinase, phosphatidylinositol 3-kinase, and protein tyrosine kinase activities (Figure 2B). KEGG pathway analysis identified the type 2 diabetes mellitus pathway as a top-enriched pathway, with JNK, PI3K, and mTOR as critical signaling nodes linking obesity to insulin resistance (Figure 2C).

### 4.3. Molecular Docking Analysis of Lyoniresinol With mTOR, PI3K, and JNK

**Figure 3.**
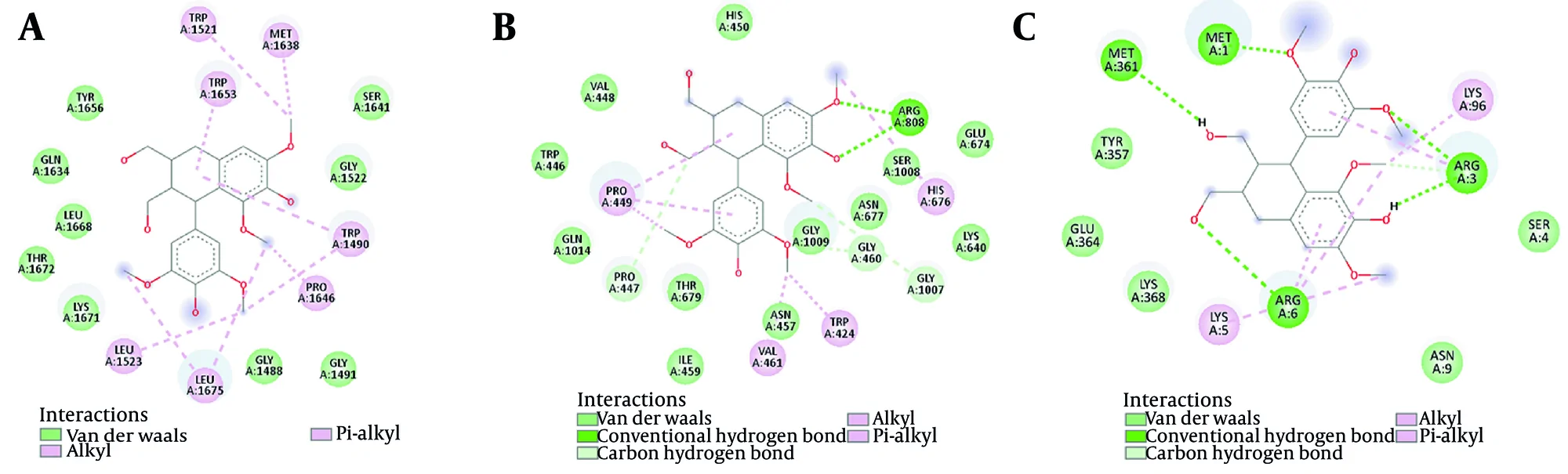
Molecular docking analysis of lyoniresinol with key target proteins. Binding models of lyoniresinol with mTOR (A), PI3K (B), and JNK (C). Key interacting residues are labeled.

Molecular docking was performed to examine the predicted binding of lyoniresinol to mTOR, PI3K, and JNK. As shown in Table 2, lyoniresinol exhibited favorable binding energies with mTOR (-6.7 kcal/mol), JNK (-7.0 kcal/mol), and PI3K (-7.5 kcal/mol). The predicted binding modes are shown in Figure 3A-C. Consistent with Table 2, the mTOR–lyoniresinol complex involved LEU A:1523, LEU A:1675, PRO A:1646, TRP A:1490, TRP A:1653, TRP A:1521, and MET A:1638; the JNK–lyoniresinol complex involved ARG A:3, ARG A:6, MET A:361, and MET A:1; and the PI3K–lyoniresinol complex involved ARG A:808. These docking results suggest potential noncovalent interactions between lyoniresinol and these signaling-related proteins.

**Table 2.** Molecular Docking Results of Lyoniresinol with Key Target Proteins

No.	Ligand	Protein	Binding Energy (kcal/mol)	Amino Acid Residues
**1**	Lyoniresinol	mTOR	-6.7	LEU A:1523, LEU A:1675, PRO A:1646, TRP A:1490, TRP A:1653, TRP A:1521, MET A:1638
**2**	Lyoniresinol	JNK	-7.0	ARG A:3, ARG A:6, MET A:361, MET A:1
**3**	Lyoniresinol	PI3K	-7.5	ARG A:808

### 4.4. Primary Endpoint: Lyoniresinol Reduces Body Weight and Adipose Tissue Mass in Obese Rats

**Figure 4.**
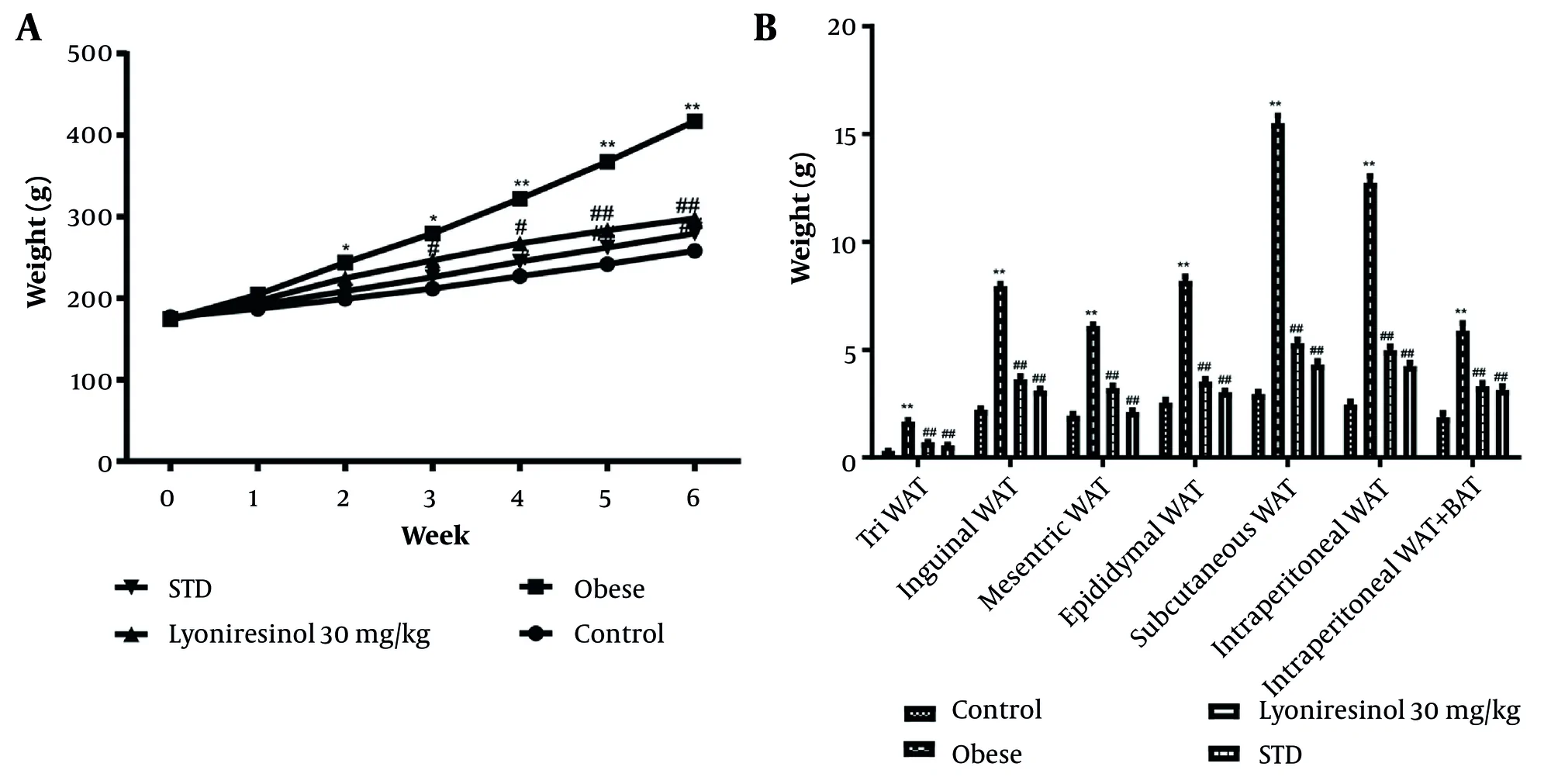
Effects of lyoniresinol on body weight and adipose tissue mass in obese rats. A: Weekly body weight (g) of rats during the 6-week experimental period. B: Weights (g) of adipose tissues from different anatomical depots at the end of the experiment. WAT, white adipose tissue. BAT, brown adipose tissue. Control, normal diet group. Obese, high-fat diet (HFD)-fed group. Lyoniresinol 30 mg/kg, HFD-fed group treated with lyoniresinol (30 mg/kg/day). STD, HFD-fed group treated with standard drug (atorvastatin, 40 mg/kg/day). Data are presented as mean ± SD (n = 6 per group). Statistical significance was determined using one-way ANOVA followed by Tukey’s multiple comparisons test. The symbols indicate statistical significance (* P < 0.05, ** P < 0.01 versus control group. # P < 0.05, ## P < 0.01 versus obese group).

As shown in Figure 4A, after six weeks of HFD feeding, the obese group exhibited a 42% increase in body weight compared with controls (P < 0.0001). Lyoniresinol treatment significantly reduced body weight gain by 18% relative to the obese group (P = 0.0046), with no significant difference between the lyoniresinol and STD groups (P = 0.2992). Figure 4B shows the combined weight of visceral, epididymal, mesenteric, and inguinal adipose tissues. Obese rats exhibited a 2.6-fold increase in total fat mass compared with controls (P = 0.0001). Lyoniresinol reduced total adipose mass by 32% relative to the obese group (P = 0.0018). These findings indicate that lyoniresinol attenuates HFD-induced body weight gain and adiposity.

### 4.5. Primary Endpoint: Lyoniresinol Ameliorates Glucose Tolerance and Insulin Sensitivity

**Figure 5.**
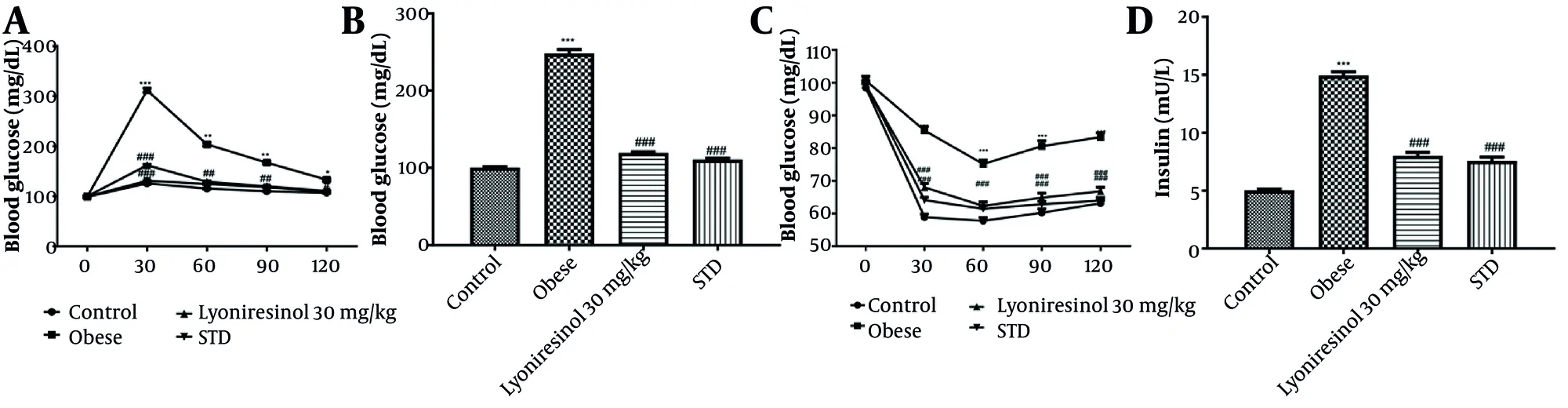
Effects of lyoniresinol on glucose homeostasis and insulin levels in obese rats. A: Oral glucose tolerance test (OGTT). Blood glucose (mg/dL) was measured at 0, 30, 60, 90, and 120 min after oral glucose administration (2 g/kg). B: Fasting blood glucose levels (mg/dL) at the end of the experimental protocol. C: Insulin tolerance test (ITT). Blood glucose (mg/dL) was measured at 0, 30, 60, 90, and 120 min after intraperitoneal insulin injection (0.75 U/kg). D: Fasting serum insulin levels (mU/L) at the end of the experimental period. Control, normal diet group. Obese, high-fat diet (HFD)-fed group. Lyoniresinol 30 mg/kg, HFD-fed group treated with lyoniresinol (30 mg/kg/day). STD, HFD-fed group treated with standard drug (atorvastatin, 40 mg/kg/day). Data are presented as mean ± SD (n = 6 per group). Statistical significance was determined using one-way ANOVA followed by Tukey’s multiple comparisons test. The symbols indicate statistical significance (* P < 0.05, ** P < 0.01, *** P < 0.001 versus control group. ## P < 0.01, ### P < 0.001 versus obese group).

In the OGTT (Figure 5A), obese rats had blood glucose levels 1.8- to 2.5-fold higher than controls at 30, 60, 90, and 120 min after glucose administration (P = 0.0061). Lyoniresinol treatment reduced glucose levels at all of these time points compared with the obese group (P = 0.0183). Fasting blood glucose (Figure 5B) was 2.2-fold higher in obese rats than in controls (P < 0.0001). Lyoniresinol lowered it by 46% (P < 0.0001 vs. obese). In the ITT (Figure 5C), obese rats showed 58% higher glucose at 60 min than controls (P < 0.0001). Lyoniresinol reduced this value by 38% (P < 0.0001 vs. obese). In addition, fasting serum insulin was 3.1-fold higher in obese rats than in controls (Figure 5D, P < 0.0001). Lyoniresinol reduced insulin by 54% (P < 0.0001 vs. obese). These data indicate that lyoniresinol ameliorates hyperglycemia, glucose intolerance, and insulin resistance in HFD-induced obese rats.

### 4.6. Key Secondary Endpoint: Lyoniresinol Induces Browning of White Adipose Tissue

**Figure 6.**
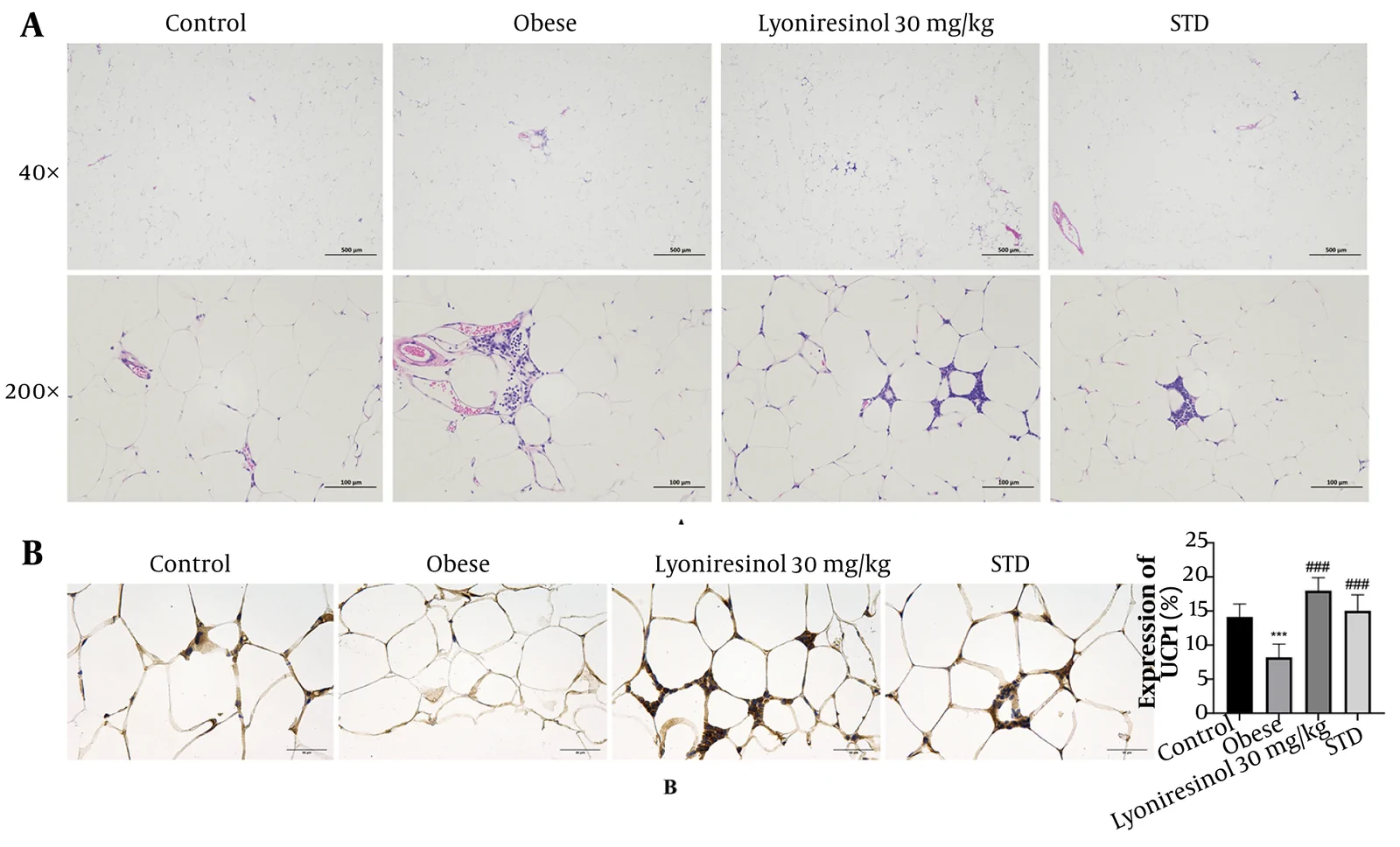
Effects of lyoniresinol on white adipose tissue morphology and UCP1 expression in obese rats. A: Representative H&E staining images of adipose tissue from different groups at 40× (scale bar: 500 μm) and 200× (scale bar: 100 μm) magnification. B: Representative immunohistochemical staining images of UCP1 in adipose tissue (200×, scale bar: 50 μm) and quantitative analysis of UCP1 expression (%). Control, normal diet group. Obese, high-fat diet (HFD)-fed group. Lyoniresinol 30 mg/kg, HFD-fed group treated with lyoniresinol (30 mg/kg/day). STD, HFD-fed group treated with standard drug (atorvastatin, 40 mg/kg/day). Data are presented as mean ± SD (n = 6 per group). Statistical significance was determined using one-way ANOVA followed by Tukey’s multiple comparisons test. The symbols indicate statistical significance (*** P < 0.001 versus control group. ### P < 0.001 versus obese group).

Histopathological changes in epididymal adipose tissue were evaluated in obese rats (Figure 6A). Control animals showed normal adipose tissue histology. In obese rats, adipose tissue exhibited pronounced hypertrophy, irregular cell morphology, and mild inflammation. Lyoniresinol treatment significantly improved these histopathological alterations and restored near-normal tissue morphology. Immunohistochemical staining revealed that UCP1 expression was markedly downregulated in the adipose tissue of obese rats compared with controls (P = 0.0002). Notably, lyoniresinol treatment significantly increased UCP1 expression relative to that in obese rats (P < 0.0001), with efficacy comparable to or greater than that of the standard drug (Figure 6B-C). These findings suggest that lyoniresinol is associated with increased UCP1 expression, consistent with adipose tissue browning.

### 4.7. Exploratory Endpoint: Lyoniresinol Ameliorates the Lipid Profile

**Figure 7.**
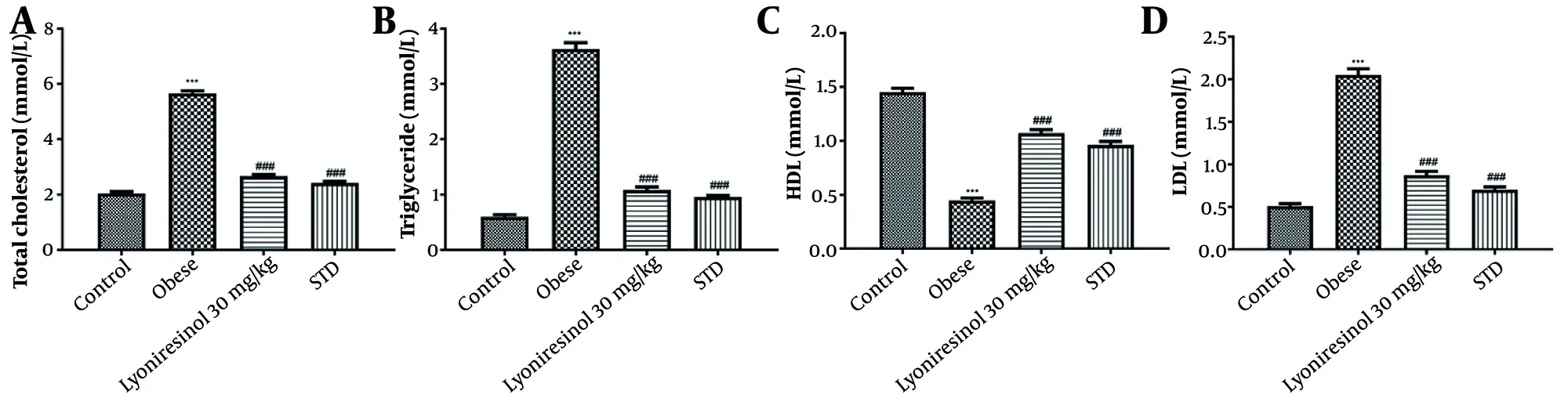
Effects of lyoniresinol on serum lipid profile in obese rats. A: Serum total cholesterol levels (mmol/L). B: Serum triglyceride levels (mmol/L). C: Serum high-density lipoprotein (HDL) levels (mmol/L). D: Serum low-density lipoprotein (LDL) levels (mmol/L). Control, normal diet group. Obese, high-fat diet (HFD)-fed group. Lyoniresinol 30 mg/kg, HFD-fed group treated with lyoniresinol (30 mg/kg/day). STD, HFD-fed group treated with standard drug (atorvastatin, 40 mg/kg/day). Data are presented as mean ± SD (n = 6 per group). Statistical significance was determined using one-way ANOVA followed by Tukey’s multiple comparisons test. The symbols indicate statistical significance (*** P < 0.001 versus control group. ### P < 0.001 versus obese group).

As shown in Figure 7A, total cholesterol was 2.3-fold higher in obese rats than in controls (P < 0.0001). Lyoniresinol treatment reduced total cholesterol by 39% compared with the obese group (P < 0.0001 vs. obese). Triglycerides (Figure 7B) were 2.8-fold higher in obese rats (P < 0.0001). Lyoniresinol lowered triglycerides by 44% (P < 0.0001 vs. obese). HDL cholesterol (Figure 7C) was 48% lower in obese rats than in controls (P < 0.0001). Lyoniresinol increased HDL by 71% relative to the obese group (P < 0.0001 vs. obese). Finally, LDL cholesterol (Figure 7D) was 3.4-fold higher in obese rats (P < 0.0001). Lyoniresinol reduced LDL by 53% (P < 0.0001 vs. obese). These data indicate that lyoniresinol reverses dyslipidemia in HFD-induced obese rats.

### 4.8. Exploratory Endpoint: Lyoniresinol Attenuates Oxidative Stress and Suppresses Inflammatory Responses

**Figure 8.**
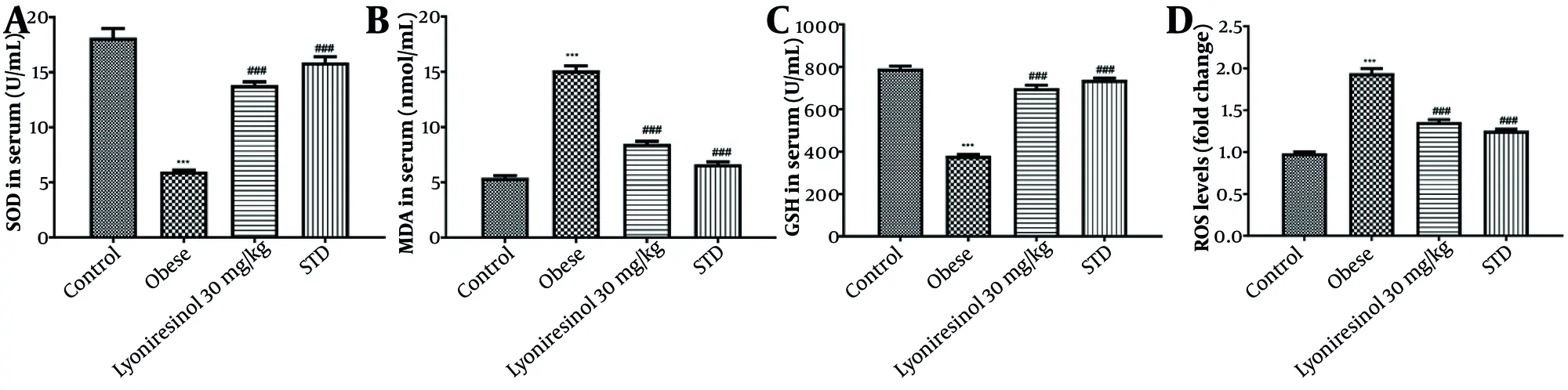
Effects of lyoniresinol on oxidative stress parameters in obese rats. A: Superoxide dismutase (SOD) activity (U/mL serum). B: Malondialdehyde (MDA) levels (nmol/mL serum). C: Glutathione (GSH) activity (U/mL serum). D: Reactive oxygen species (ROS) levels (fold changes in fluorescence intensity). Control, normal diet group. Obese, high-fat diet (HFD)-fed group. Lyoniresinol 30 mg/kg, HFD-fed group treated with lyoniresinol (30 mg/kg/day). STD, HFD-fed group treated with standard drug (atorvastatin, 40 mg/kg/day). Data are presented as mean ± SD (n = 6 per group). Statistical significance was determined using one-way ANOVA followed by Tukey’s multiple comparisons test. The symbols indicate statistical significance (*** P < 0.001 versus control group. ### P < 0.001 versus obese group).

**Figure 9.**
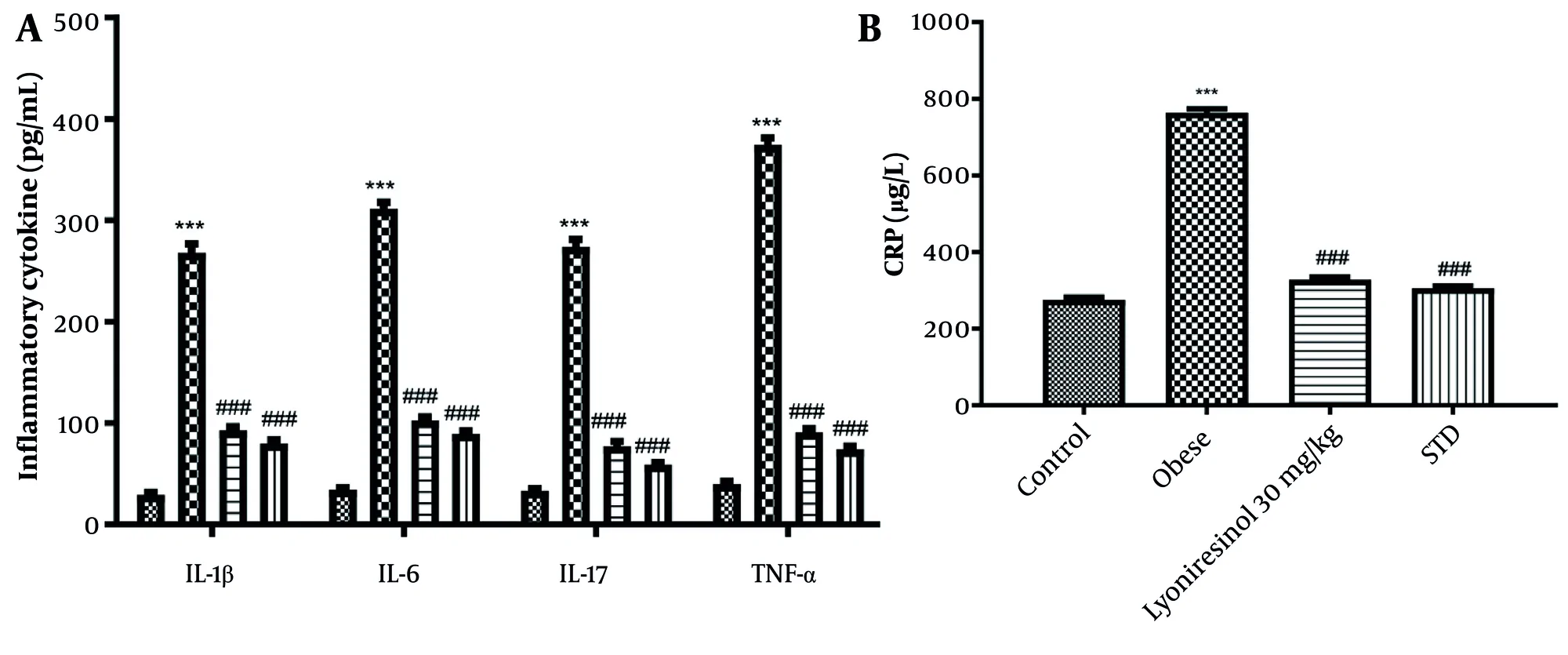
Effects of lyoniresinol on inflammatory mediators in obese rats. A: Serum levels of IL-1β, IL-6, IL-17, and TNF-α. B: Serum levels of C-reactive protein (CRP) levels. Control, normal diet group. Obese, high-fat diet (HFD)-fed group. Lyoniresinol 30 mg/kg, HFD-fed group treated with lyoniresinol (30 mg/kg/day). STD, HFD-fed group treated with standard drug (atorvastatin, 40 mg/kg/day). Data are presented as mean ± SD (n = 6 per group). Statistical significance was determined using one-way ANOVA followed by Tukey’s multiple comparisons test. The symbols indicate statistical significance (*** P < 0.001 versus control group. ### P < 0.001 versus obese group).

As shown in Figure 8A, SOD activity was 2.4-fold lower in obese rats than in controls (P < 0.0001). Lyoniresinol treatment increased SOD activity to 2.0-fold of that in the obese group (P < 0.0001 vs. obese). MDA levels (Figure 8B) were 3.1-fold higher in obese rats (P < 0.0001). Lyoniresinol reduced MDA to 0.54-fold of that in the obese group (P < 0.0001 vs. obese). GSH levels (Figure 8C) were 0.33-fold lower in obese rats than in controls (P < 0.0001). Lyoniresinol increased GSH to 2.8-fold of that in the obese group (P < 0.0001 vs. obese). Finally, ROS levels (Figure 8D) were 2.7-fold higher in obese rats (P < 0.0001). Lyoniresinol reduced ROS to 0.59-fold of that in the obese group (P < 0.001 vs. obese). Serum levels of inflammatory mediators were also evaluated (Figure 9). Obese rats had elevated levels of IL-1β, IL-6, IL-17, TNF-α, and CRP compared with controls. Lyoniresinol treatment significantly reduced these elevated cytokine and CRP levels. These data indicate that lyoniresinol alleviates oxidative stress and inflammatory responses in HFD-induced obese rats.

### 4.9. Mechanistic Validation: Lyoniresinol Is Associated With Altered JNK/PI3K/mTOR Phosphorylation

**Figure 10.**
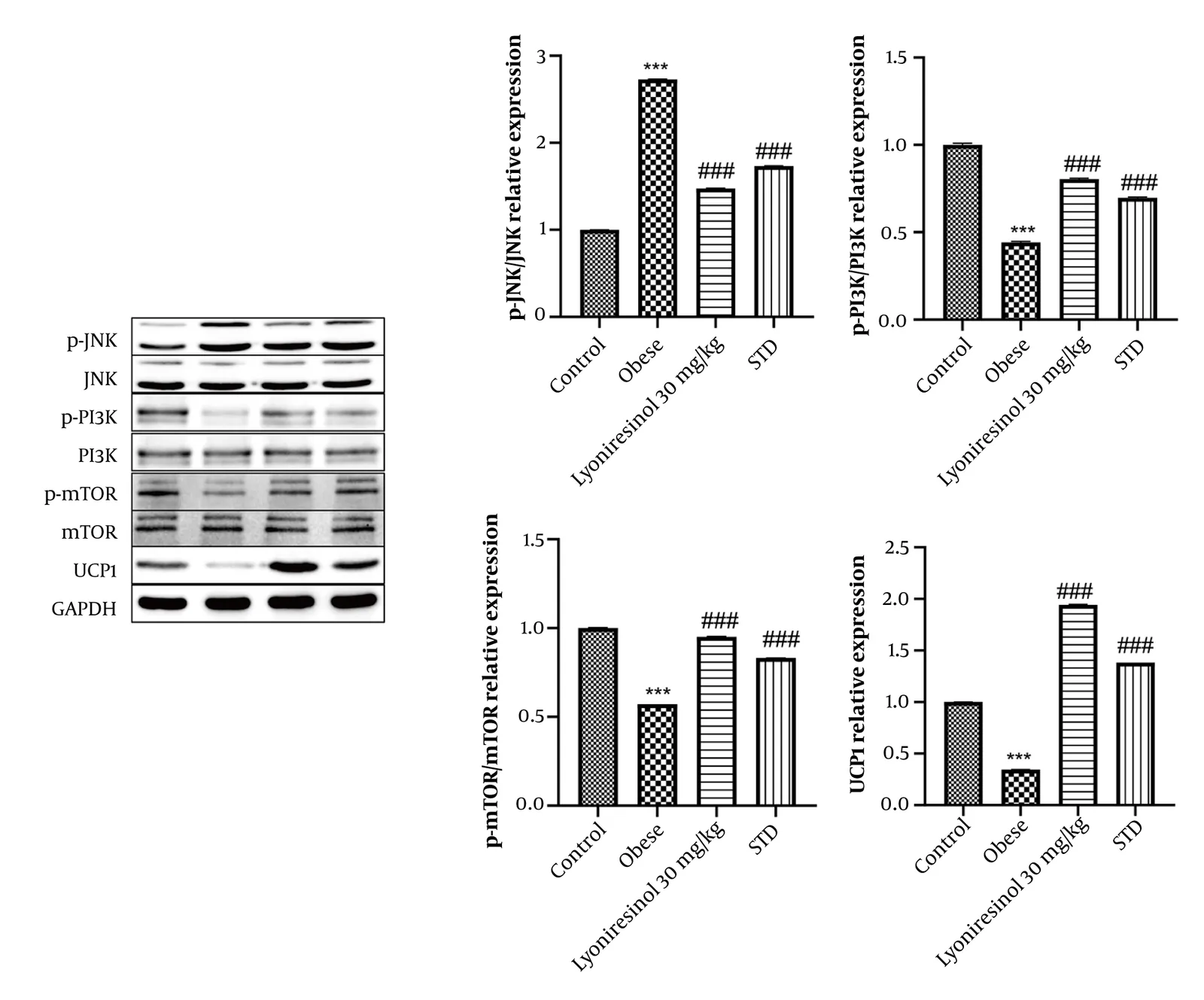
Effects of lyoniresinol on the JNK/PI3K/mTOR signaling pathway in obese rats. Representative Western blot images and quantitative analysis of phosphorylated JNK (p-JNK), total JNK, phosphorylated PI3K (p-PI3K), total PI3K, phosphorylated mTOR (p-mTOR), total mTOR, and UCP1 protein expression levels. Phosphorylated protein levels were quantified relative to their corresponding total proteins (p-JNK/JNK, p-PI3K/PI3K, and p-mTOR/mTOR). Total JNK, PI3K, mTOR, and UCP1 protein expression levels were normalized to GAPDH. Control, normal diet group. Obese, high-fat diet (HFD)-fed group. Lyoniresinol 30 mg/kg, HFD-fed group treated with lyoniresinol (30 mg/kg/day). STD, HFD-fed group treated with standard drug (atorvastatin, 40 mg/kg/day). Data are presented as mean ± SD (n = 6 per group). Statistical significance was determined using one-way ANOVA followed by Tukey’s multiple comparisons test. The symbols indicate statistical significance (*** P < 0.001 versus control group. ### P < 0.001 versus obese group).

We examined the effects of lyoniresinol on JNK/PI3K/mTOR pathway proteins and UCP1 expression in adipose tissue (Figure 10). Western blot analysis revealed elevated p-JNK and reduced p-PI3K, p-mTOR, and UCP1 expression levels in obese rats relative to controls (all P < 0.0001). Lyoniresinol treatment markedly downregulated p-JNK expression and upregulated p-PI3K, p-mTOR, and UCP1 levels (all P < 0.0001). These results suggest that lyoniresinol treatment was associated with altered JNK/PI3K/mTOR phosphorylation together with increased UCP1 expression.

## 5. Discussion

Obesity contributes to chronic conditions such as type 2 diabetes and typically results from energy intake exceeding energy expenditure. Recent epidemiological data indicate that approximately 350 million children and adolescents aged 5 - 19 years are affected by obesity, and the prevalence is projected to continue rising (17). Although several anti-obesity medications have demonstrated substantial clinical efficacy, adverse effects and concerns regarding long-term safety have limited their broader clinical use, underscoring the need for safer and more effective therapeutic approaches (15, 16).

In this study, we used network pharmacology to explore the potential molecular mechanisms of lyoniresinol against obesity. We identified 101 targets of lyoniresinol, of which 50 overlapped with obesity-related genes. Functional enrichment analysis showed that the core targets associated with lyoniresinol activity were enriched in type 2 diabetes-related signaling pathways, including PI3K, JNK, and mTOR, suggesting their potential involvement in obesity-related metabolic regulation. Furthermore, molecular docking predicted favorable interactions between lyoniresinol and these three key proteins, with binding energies of -6.7, -7.5, and -7.0 kcal/mol, respectively. Collectively, the network pharmacology and docking analyses suggest that PI3K, JNK, and mTOR may represent potential molecular targets of lyoniresinol, providing a basis for subsequent experimental investigation.

Rats fed a HFD developed clinical features resembling human obesity (12). HFD consumption increased body weight, a hallmark of obesity, consistent with previous reports. Effective obesity management is typically defined by a reduction in body weight to near-normal levels (17). In the present study, lyoniresinol-treated rats showed lower body weight than untreated obese rats. Obesity is strongly linked to type 2 diabetes, and increased fat deposition in adipocytes disrupts lipid metabolism. Chronic HFD administration is a well-known cause of hyperlipidemia (15). In our study, HFD-fed rats exhibited marked increases in serum cholesterol, triglycerides, LDL, and VLDL levels and a decrease in HDL compared with controls. These lipid alterations were reversed by lyoniresinol treatment.

Oxidative stress is an underlying factor in chronic diseases, including obesity. Accumulating evidence shows that HFD intake and high lipid levels impair cellular metabolism, increasing ROS and free radical generation (22). This rise in ROS leads to increased superoxide anion production, which is associated with increased lipid peroxidation and membrane damage, as reflected by elevated MDA levels. Superoxide anions are normally metabolized by SOD. However, SOD activity is compromised in obesity, further aggravating oxidative stress (25). Reduced GSH levels have also been reported in obesity. Oxidative stress-induced cellular damage is linked to increased production of inflammatory cytokines. In the present study, obese rats showed higher oxidative stress markers than controls. The literature indicates that reducing oxidative stress can restore metabolic regulation and limit fat accumulation, thereby helping to manage obesity (21). Lyoniresinol treatment reversed the altered oxidative stress parameters in obese rats.

Oxidative stress-induced cellular injury is thought to trigger inflammatory mediators such as IL-17, IL-6, and CRP, which can alter metabolic cascades and promote obesity (22). Spirulina supplementation has also been shown to regulate inflammatory pathways, including NLRP3, in obese adults, supporting the potential of natural compounds to modulate obesity-related inflammation (26). In addition, inflammatory changes during adipose tissue remodeling modulate adipocyte function, enhancing fat deposition and WAT formation (24). In our study, inflammatory cytokine levels were higher in obese rats than in controls, whereas lyoniresinol treatment was associated with suppression of these increases. Adipose tissue exists as two main types: BAT and WAT. BAT contains small lipid droplets and abundant mitochondria and is involved in thermogenesis (28). In contrast, WAT contains large lipid droplets, has reduced mitochondrial content, and exhibits impaired insulin receptor sensitivity. Lipid droplets in WAT prevent glucose transporter translocation to the cell membrane, leading to decreased glucose utilization (29). This reduction increases blood glucose levels, which, through negative feedback, elevates serum insulin. Consistent with this, obese rats in our study had higher adipose tissue weight, blood glucose, and serum insulin levels than controls.

Obesity is a major cause of type 2 diabetes, and our GO and KEGG analyses linked obesity-related genes to type 2 diabetes. We observed that lyoniresinol treatment was associated with decreased p-JNK and increased p-PI3K and p-mTOR levels in the adipose tissue of obese rats, suggesting potential modulation of stress-related and metabolic signaling pathways. Previous reports have shown that obesity is associated with activation of JNK and dysregulation of PI3K/mTOR, contributing to insulin resistance (27). Obesity also increases ROS formation, which activates JNK and affects insulin secretion. The PI3K pathway has been pharmacologically modulated to ameliorate obesity (31). Consistent with these reports, our data showed that lyoniresinol treatment not only correlated with correction of the imbalance in JNK and PI3K/mTOR phosphorylation but also was associated with significantly upregulated UCP1 expression in adipose tissue. Aerobic training combined with stevia supplementation has similarly been reported to influence glucose metabolism signaling pathways in the cardiac tissue of obese rats, suggesting that combined lifestyle and phytochemical interventions may offer synergistic benefits for metabolic regulation (32). Upregulation of UCP1 suggests that lyoniresinol may promote the browning of white adipose tissue, a process that enhances energy expenditure and thermogenesis. Epididymal white adipose tissue (eWAT) was analyzed in the present study because it represents a major visceral adipose depot closely associated with obesity-related metabolic dysfunction, insulin resistance, and inflammation (33). Therefore, assessment of histological changes and UCP1 expression in eWAT provided relevant evidence regarding the metabolic effects of lyoniresinol. Future studies incorporating both visceral and subcutaneous adipose depots will be necessary to determine whether lyoniresinol exhibits depot-dependent browning effects. Taken together, our findings indicate that lyoniresinol improves insulin sensitivity and is associated with changes suggestive of WAT browning. The concurrent alterations in JNK and PI3K/mTOR phosphorylation suggest the potential involvement of these signaling pathways in the observed metabolic effects. However, these findings do not establish a direct causal relationship.

### 5.1. Limitations

Several limitations should be acknowledged. First, although molecular docking provides useful information on potential compound-target interactions, it cannot fully reflect the dynamic behavior of protein-ligand complexes. Further validation using molecular dynamics simulations and experimental studies is needed. Second, although lyoniresinol treatment was accompanied by changes in JNK, PI3K, and mTOR phosphorylation and enhanced UCP1 expression, our findings do not demonstrate that these signaling alterations are directly responsible for the metabolic benefits. Because we did not apply pharmacological inhibition or genetic manipulation of these pathways, the functional contribution of JNK/PI3K/mTOR signaling requires further confirmation. Future studies employing pathway-specific interventions will help clarify the molecular mechanisms involved. Third, our analysis was limited to epididymal white adipose tissue. Future studies should also examine inguinal white adipose tissue to determine whether lyoniresinol induces depot-specific browning effects.

### 5.2. Conclusions

In conclusion, lyoniresinol treatment was associated with improved insulin sensitivity and reduced adiposity in HFD-induced obese rats, meeting the predefined primary efficacy endpoints. Network pharmacology analysis suggested that PI3K, JNK, and mTOR may represent key targets associated with the biological effects of lyoniresinol, while molecular docking analysis indicated potential interactions between lyoniresinol and these proteins. These findings, together with the observed alterations in JNK/PI3K/mTOR phosphorylation and increased UCP1 expression, suggest that this signaling axis may contribute to the metabolic effect of lyoniresinol. However, further studies using pathway-specific interventions are required to establish causal relationships. Additional exploratory analyses indicated potential improvements in lipid metabolism, inflammation, and oxidative stress. Overall, lyoniresinol represents a promising candidate for further investigation as a multi-target therapeutic agent for obesity and related metabolic disorders.

## Data Availability

Data will be made available on request from the corresponding author.

## References

[1] Jin X, Qiu T, Li L, Yu R, Chen X, Li C (2023). Pathophysiology of obesity and its associated diseases. Acta Pharm Sin B.

[2] Chand S, Tripathi AS, Dewani AP, Sheikh NWA (2024). Molecular targets for management of diabetes: Remodelling of white adipose to brown adipose tissue. Life Sci.

[3] Yan K (2024). Recent advances in the effect of adipose tissue inflammation on insulin resistance. Cell Signal.

[4] Saghebjoo M, Seyed Moosavi SM, Ghasemi E, Hedayati M (2025). The Effect of Endurance Training and Dill Seed Extract on Insulin Sensitivity and Nicotinamide N-methyltransferase Levels in the Liver and Adipose Tissue of Obese Rats. Jundishapur JNatPharm Prod.

[5] Wen X, Zhang B, Wu B, Xiao H, Li Z, Li R (2022). Signaling pathways in obesity: mechanisms and therapeutic interventions. Signal Transduct Target Ther.

[6] Díaz ME, González L, Miquet JG, Martínez CS, Sotelo AI, Bartke A (2012). Growth hormone modulation of EGF-induced PI3K-Akt pathway in mice liver. Cell Signal.

[7] Huang X, Liu G, Guo J, Su Z (2018). The PI3K/AKT pathway in obesity and type 2 diabetes. Int J Biol Sci.

[8] Wang L, Li J, Di L (2022). Glycogen synthesis and beyond, a comprehensive review of GSK3 as a key regulator of metabolic pathways and a therapeutic target for treating metabolic diseases. Med Res Rev.

[9] Hinoi E, Iezaki T, Fujita H, Watanabe T, Odaka Y, Ozaki K (2014). PI3K/Akt is involved in brown adipogenesis mediated by growth differentiation factor-5 in association with activation of the Smad pathway. Biochem Biophys Res Commun.

[10] Isah MB, Tajuddeen N, Yusuf A, Mohammed A, Ibrahim MA, Melzig M (2025). The antidiabetic properties of lignans: a comprehensive review. Phytomedicine.

[11] Kanchanapoom T, Kamel MS, Kasai R, Yamasaki K, Picheansoonthon C, Hiraga Y (2001). Lignan glucosides from Acanthus ilicifolius. Phytochemistry.

[12] Tomosaka H, Chin Y, Salim AA, Keller WJ, Chai H, Kinghorn AD (2008). Antioxidant and cytoprotective compounds from Berberis vulgaris (barberry). Phytother Res.

[13] Liu H, Sui X, Li X, Li Y (2013). Lyoniresinol inhibits melanogenic activity through the induction of microphthalmia-associated transcription factor and extracellular receptor kinase activation. Mol Cell Biochem.

[14] Guo XH, Pang L, Gao CY, Meng FL, Jin W (2023). Lyoniresinol attenuates cerebral ischemic stroke injury in MCAO rat based on oxidative stress suppression via regulation of Akt/GSK-3β/Nrf2 signaling. Biomed Pharmacother.

[15] Chand S, Tripathi AS, Hasan T, Ganesh K, Cordero MAW, Yasir M (2023). Geraniol reverses obesity by improving conversion of WAT to BAT in high fat diet induced obese rats by inhibiting HMGCoA reductase. Nutr Diabetes.

[16] Ansari S, Khoo B, Tan T (2024). Targeting the incretin system in obesity and type 2 diabetes mellitus. Nat Rev Endocrinol.

[17] Jebeile H, Kelly AS, O'Malley G, Baur LA (2022). Obesity in children and adolescents: epidemiology, causes, assessment, and management. Lancet Diabetes Endocrinol.

[18] Shi Q, Wang Y, Hao Q, Vandvik PO, Guyatt G, Li J (2024). Pharmacotherapy for adults with overweight and obesity: a systematic review and network meta-analysis of randomised controlled trials. Lancet.

[19] Kelly AS (2023). Current and future pharmacotherapies for obesity in children and adolescents. Nat Rev Endocrinol.

[20] Hall KD, Kahan S (2018). Maintenance of Lost Weight and Long-Term Management of Obesity. Med Clin North Am.

[21] Romieu I, Dossus L, Barquera S, Blottière HM, Franks PW, Gunter M (2017). Energy balance and obesity: what are the main drivers?. Cancer Causes Control.

[22] Manna P, Jain SK (2015). Obesity, Oxidative Stress, Adipose Tissue Dysfunction, and the Associated Health Risks: Causes and Therapeutic Strategies. Metab Syndr Relat Disord.

[23] Andrés CMC, Pérez de la Lastra JM, Andrés Juan C, Plou FJ, Pérez-Lebeña E (2023). Superoxide Anion Chemistry-Its Role at the Core of the Innate Immunity. Int J Mol Sci.

[24] Tobore TO (2020). Towards a comprehensive theory of obesity and a healthy diet: The causal role of oxidative stress in food addiction and obesity. Behav Brain Res.

[25] Čolak E, Pap D (2021). The role of oxidative stress in the development of obesity and obesity-related metabolic disorders. J Med Biochem.

[26] Farshad A, Farshad G, Mahdi S, Hossein K, Mandana G (2022). Spirulina for Protection Against COVID-19 via Regulating ACE2, FNDC5, and NLRP3: A Triple-Blind Randomized Placebo-Controlled Trial in Obese Adults: Spirulina for protection against COVID-19. J Cell Mol Anesth.

[27] Kawai T, Autieri MV, Scalia R (2021). Adipose tissue inflammation and metabolic dysfunction in obesity. Am J Physiol Cell Physiol.

[28] Cedikova M, Kripnerová M, Dvorakova J, Pitule P, Grundmanova M, Babuska V (2016). Mitochondria in White, Brown, and Beige Adipocytes. Stem Cells Int.

[29] Santoro A, McGraw TE, Kahn BB (2021). Insulin action in adipocytes, adipose remodeling, and systemic effects. Cell Metab.

[30] McArdle MA, Finucane OM, Connaughton RM, McMorrow AM, Roche HM (2013). Mechanisms of obesity-induced inflammation and insulin resistance: insights into the emerging role of nutritional strategies. Front Endocrinol (Lausanne).

[31] Savova MS, Mihaylova LV, Tews D, Wabitsch M, Georgiev MI (2023). Targeting PI3K/AKT signaling pathway in obesity. Biomed Pharmacother.

[32] Zahra A, Asieh AD, Ahmad A, Sayed JZ (2023). The effect of aerobic training and stevia supplement on expression of the glucose metabolism signaling pathway markers in heart tissue of obese rats. J Semnan Univ Med Sci.

[33] Franczyk MP, He M, Yoshino J (2021). Removal of Epididymal Visceral Adipose Tissue Prevents Obesity-Induced Multi-organ Insulin Resistance in Male Mice. J Endocr Soc.

